# A System of Midwifery

**Published:** 1874-01

**Authors:** 


*A System of Midwifery; including the Diseases of Pregnancy and Puerperal State.* By William Lieshman, M. D., Regius Professor of Midwifery in the University of Glasgow. Philadelphia: Henry C. Lea, 1873.

